# Physical and mental fatigue in people with non-communicable chronic diseases

**DOI:** 10.1080/07853890.2022.2122553

**Published:** 2022-09-16

**Authors:** Anouk W. Vaes, Yvonne M. J. Goërtz, Maarten van Herck, Rosanne J. H. C. G. Beijers, Martijn van Beers, Chris Burtin, Daisy J. A. Janssen, Annemie M. W. J. Schols, Martijn A. Spruit

**Affiliations:** a Department of Research and Development, Ciro, Horn, The Netherlands; b Department of Respiratory Medicine, Nutrim School of Nutrition and Translational Research in Metabolism, Maastricht University, Maastricht, The Netherlands; c REVAL Rehabilitation Research Center, BIOMED Research Institute, Faculty of Rehabilitation Sciences, Hasselt University, Diepenbeek, Belgium; d Department of Health Services Research, Care and Public Health Research Institute, Faculty of Health, Medicine and Life Sciences, Maastricht University, Maastricht, The Netherlands

**Keywords:** Fatigue, chronic diseases, physical fatigue, mental fatigue

## Abstract

**Background:**

Fatigue is frequently reported in people with a non-communicable chronic disease. More insight in the nature of this symptom may enhance targeted treatment of fatigue. In this study, we aimed to gain more insight in the prevalence of different types of fatigue and in current prescribed treatment strategies to reduce fatigue in non-communicable chronic diseases.

**Methods:**

People with non-communicable chronic diseases were contacted *via* public, non-profit, disease-specific health funds and patient associations and invited to complete a web-based survey. The survey included a general question about the experience (“Do you now or have you ever had complaints of fatigue?”) and nature of fatigue (physically/mentally/combination), the Checklist Individual Strength-subscale subjective fatigue (CIS-Fatigue; 8–56 points), self-constructed questions for the distinction between physical and mental fatigue (both 3–21 points) and questions on prescribed treatments for fatigue.

**Results:**

In total, 4199 participants (77% females) completed the online survey. 3945 participants (94.0%) reported experiencing fatigue, of which 64.4% reported a combination of both physical and mental fatigue. Median CIS-Fatigue score was 41 (32–48) points, with 68% of the participants reporting severe fatigue (≥36 points). Median scores for physical and mental fatigue were 15 (11–18) and 12 (8–16) points, respectively. In 55% of the participants, fatigue was only occasionally or never discussed with the healthcare professional, and only 23% of the participants were prescribed a treatment for fatigue. Participants often reported no effect or even an increase in fatigue after treatment.

**Conclusions:**

Findings indicate that both physical and mental fatigue are often experienced simultaneously in people with non-communicable chronic diseases, but can also occur separately. Fatigue is often only occasionally or never discussed, let alone treated, highlighting the need to raise awareness among healthcare professionals. Future studies are needed to gain more insight in underlying factors of fatigue in non-communicable chronic diseases, its impact on daily life and development and evaluation of targeted treatment strategies.

**Key messages:**
- Both physical and mental fatigue are frequently present in people with non-communicable chronic diseases.
- Fatigue is often only occasionally or never discussed during consultation with the physician, highlighting the need to raise awareness among healthcare professionals for adequate screening and evaluating of fatigue in people with non-communicable chronic diseases.
- Only less than a quarter of the people with non-communicable chronic diseases who reported to experience fatigue were prescribed a treatment for fatigue, which was often experienced as ineffective.

## Introduction

Fatigue is defined as “a subjective, unpleasant symptom which incorporates total body feelings ranging from tiredness to exhaustion creating an unrelenting overall condition which interferes with individuals’ ability to function to their normal capacity” [1]. It has been recognized that fatigue is a multidimensional construct, including a physical and mental component [2]. Physical fatigue is characterized by difficulties in performing physical activities, while mental/cognitive fatigue is described as difficulties concentrating and performing cognitive tasks [2]. As fatigue is a subjective experience, it is difficult to measure and a gold standard is lacking. A range of fatigue assessment scales are available, including generic or disease-specific scales, unidimensional scales assessing the severity of fatigue and multidimensional scales covering different dimensions of fatigue, such as severity, duration, nature and impact of fatigue [3].

Fatigue is a frequently present symptom among people with non-communicable chronic diseases. Earlier studies demonstrated prevalence rates for severe fatigue ranging from around 25% in inflammatory bowel diseases, up to 50% in respiratory diseases and 60% in brain diseases and chronic kidney diseases [4–7], and it has been shown to have a serious impact on people’s daily lives [4–6,8–13]. Indeed, it affects their ability to perform activities of daily life, such as personal care, household chores, work and socializing and contributes to an impaired quality of life [9]. Although it is affirmed that fatigue is a multidimensional construct, earlier studies of fatigue in non-communicable chronic diseases mainly focussed on the physical dimension of fatigue [14].

It has been recognized that fatigue can best be explained by an interplay between biological, psychological and social factors, and these factors are likely to be trans-diagnostic [4,5,15].

To date, several fatigue management strategies are used, including cognitive behavioural therapy, exercise therapy, medication or advice on sleep. However, data on the effectiveness of these interventions are conflicting, presenting challenges for the health care professionals in prescribing treatments for patients with non-communicable chronic diseases [16]. This study aimed to: 1) provide more insight in the prevalence of general, physical and mental fatigue in people with common non-communicable chronic diseases; and 2) to evaluate the prescribed treatment strategies to reduce fatigue in people with non-communicable chronic diseases.

## Methods

### Study design and participants

In this prospective study, people with non-communicable chronic diseases were invited to complete a web-based survey administered using Asolutions between 1 October and 30 November 2020. Participants were contacted *via* public, non-profit, disease-specific health funds and patient associations (*n* = 14; Dutch Heart Foundation (Hartstichting), Dutch Arthritis Society (ReumaNederland), Lung Foundation Netherlands (Longfonds), Dutch Kidney Foundation (Nierstichting), Dutch Diabetes Foundation (Diabetes Fonds), Princess Beatrix Muscle Foundation (Prinses Beatrix Spierfonds), Dutch Neuromuscular Disease Association (Spierziekten Nederland), Dutch Digestive Disease Foundation (Maag Lever Darm Stichting), Dutch Brain Foundation (Hersenstichting), Dutch Foundation for Mental Health (MIND), Dutch Burn Foundation (Brandwonden Stichting), Dutch myalgic encephalomyelitis/chronic fatigue syndrome (ME/CFS) Foundation (ME/CVS Stichting), Dutch Patient Association for Cardiovascular Diseases (Harteraad) and Irritable Bowel Syndrome Patient Association (Prikkelbare Darm Syndroom Belangenorganisatie)). No exclusion criteria were applied.

Ethical approval for this study was waived by the medical ethics committee of the University Hospital Maastricht and Maastricht University (METC azM/UM) because the Medical Research Involving Human Subjects Act (WMO) does not apply to this study (METC 2019-1225). Digital informed consent was obtained from all respondents at the start of the survey. This study was registered at the Netherlands Trial Register (NL8742).

### Measures

#### Demographics and disease-specific characteristics

The survey contained questions regarding sociodemographic characteristics, including sex, age, ethnicity, marital status, presence of children, education level and work status and disease- specific characteristics.

#### General, physical and mental fatigue

Fatigue was assessed using a general question about the experience (“Do you now or have you ever had complaints of fatigue?”) and nature of fatigue (physically/mentally/combination). In addition, participants were asked to fill out the Checklist Individual Strength Subscale subjective fatigue (CIS-Fatigue), which is a reliable and well-validated measure of fatigue and already applied in different non-communicable diseases [5,17–19]. The CIS-Fatigue consists of eight items scored on seven-point Likert scale. Scores range from 8 to 56 points, with higher scores indicating more clinical symptoms of general fatigue. Based upon validated cut-off values, individuals can be classified as having normal (≤26 points), mild (27–35 points), and severe fatigue (≥36 points) [17–19]. Three questions of the CIS-Fatigue questionnaire were used to evaluate self-constructed physical fatigue (“Physically I feel exhausted”, “Physically I feel I am in a bad condition” and “Physically I feel in a good shape”). In addition, three questions were constructed (by replacing the word “physically” by “mentally”) to evaluate mental fatigue (“Mentally I feel exhausted”, “Mentally I feel I am in a bad condition” and “Mentally I feel in a good shape”), as was done before in patients with long COVID [20]. The physical and mental fatigue questions were scored on a seven-point Likert scale, with scores ranging from 3 to 21 points. A higher score indicates worse physical and mental fatigue, respectively. Scores of 5 and higher on the different subitems were used as cut-offs to identify participants feeling physically/mentally exhausted or feeling physically/mentally in a bad conditions.

#### Prescribed treatment strategies

Participants who reported to experience fatigue were asked about the extent to which fatigue was discussed with the general practitioner or medical specialist (never, once, rarely, occasionally, regularly and every time), the treatments that were prescribed for their fatigue and the effectiveness of these treatments upon fatigue.

The complete questionnaire is included in Appendix 1.

### Statistical analyses

Data are presented as median and interquartile ranges for continuous data or as frequencies and proportions for categorical data. As earlier studies suggested higher fatigue scores in females, people aged <60 years and people living alone [21–23], between-group analyses in fatigue scores were performed with Mann–Whitney U Tests for sex (male *vs.* female), age (<61 years *vs.* ≥61 years old) and marital status (living together *vs.* living alone). Differences in physical and mental fatigue scores between patient associations were tested using Kruskal–Wallis tests. Statistical analyses were conducted using SPSS version 25.0 (IBM Corporation, Armonk, NY). A priori, the level of significance was set at *p* < .05.

## Results

### Participant characteristics

Participant characteristics are listed in Table 1. In total, 4199 participants completed the online survey. About three quarter of the participants were females and more than half of the participants were aged between 51 and 70 years old. Seventy percent of the participants were married or living with a partner, and 56% had children living at home. The majority of the participants did not have a paid employment. The large majority of the participants had received the link to web-based survey from the Dutch Arthritis Society (49.1%), but we also included participants from patient associations for heart diseases (16.4%), digestive diseases (8.5%), neuromuscular diseases (6.5%), ME/CFS (6.0%), lung diseases (4.0%), kidney diseases (3.8%), burns (2.5%), diabetes (1.7%), mental diseases (1.0%) or brain diseases (0.7%). See Supplementary Table 1 for the characteristics of these subgroups.

**Table 1.** Participant characteristics.

	All participants (*n* = 4199)
Sex, *n* (%)	
Male	951 (22.6)
Female	3239 (77.1)
Other/I do not want to say	9 (0.2)
Age, years, *n* (%)	
18–30	241 (5.7)
31–40	339 (8.1)
41–50	653 (15.6)
51–60	1131 (26.9)
61–70	1221 (29.1)
71–80	563 (13.4)
≥81	51 (1.2)
Ethnicity, *n* (%)	
White	4065 (96.8)
Hispanic/Latino	10 (0.2)
Black/African American	12 (0.3)
Asian/Pacific Islander	60 (1.4)
Other/I do not want to say	52 (1.2)
Marital status, *n* (%)	
Living alone	832 (19.8)
Married/living together	2937 (69.9)
Divorced	257 (6.1)
Widow/widower	173 (4.1)
Children, *n* (%)	
Yes	2769 (65.9)
* Children living at home*	1555 (56.2)
Education level, *n* (%)	
Low	886 (21.1)
Moderate	1575 (37.5)
High	1738 (41.4)
Work situation, *n* (%)	
Full-time job	435 (10.4)
Part-time job	1166 (27.8)
No paid employment	2598 (61.9)
Questionnaire received *via*, *n* (%)	
Dutch Heart Foundation (Hartstichting)	544 (13.0)
Dutch Arthritis Society (ReumaNederland)	2060 (49.1)
Lung Foundation Netherlands (Longfonds)	167 (4.0)
Dutch Kidney Foundation (Nierstichting)	159 (3.8)
Dutch Diabetes Foundation (Diabetes Fonds)	73 (1.7)
Princess Beatrix Muscle Foundation (Prinses Beatrix Spierfonds)	58 (1.4)
Dutch Neuromuscular Disease Association (Spierziekten Nederland)	213 (5.1)
Dutch Digestive Disease Foundation (Maag Lever Darm Stichting)	337 (8.0)
Dutch Brain Foundation (Hersenstichting)	28 (0.7)
Dutch Foundation for Mental Health (MIND)	43 (1.0)
Dutch Burn Foundation (Brandwonden Stichting)	103 (2.5)
Dutch ME/CFS Foundation (ME/CVS Stichting)	250 (6.0)
Dutch Patient Association for Cardiovascular Diseases (Harteraad)	142 (3.4)
Irritable Bowel Syndrom Patient Association (Prikkelbare Darm Syndroom Belangenorganisatie)	22 (0.5)

### General, physical and mental fatigue

A total of 3945 participants (94%) reported experiencing fatigue, of which the majority (64.4%) indicated to experience a combination of both physical and mental fatigue (Table 2).

**Table 2.** Fatigue-related outcomes.

	All participants (*n* = 4199)
Experiencing fatigue, *n* (%)	
No	254 (6.0)
Yes	3945 (94.0)
Mainly physical fatigue^a^	1299 (32.9)
Mainly mental fatigue^a^	106 (2.7)
Both physical and mental fatigue^a^	2540 (64.4)
General fatigue (CIS-Fatigue), median (IQR)	41 (32–48)
Severe fatigue, *n* (%)	2853 (67.9)
Physical fatigue, median (IQR)	15 (11–18)
Mental fatigue, median (IQR)	12 (8–16)

^a^Based on 3945 patients experiencing fatigue.

*n*: number; CIS-Fatigue: checklist individual strength-subscale subjective fatigue; IQR: interquartile range

**Figure 1.**
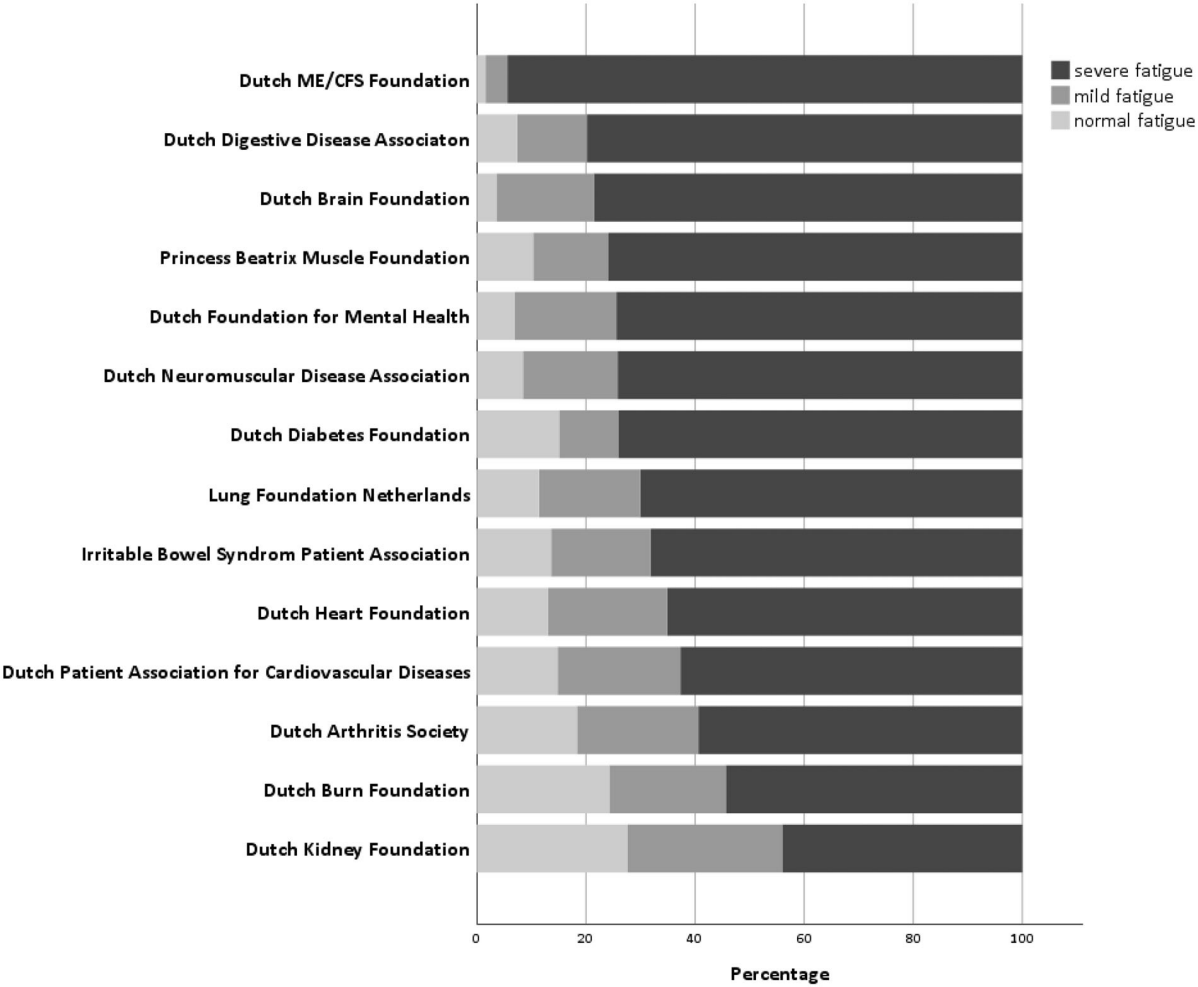
Fatigue severity in non-communicable diseases stratified by patient association (*n* = 4199). Normal (≤26 points), mild (27–35 points) and severe fatigue (≥36 points) on CIS-Fatigue.

Participants had a median CIS-Fatigue score of 41 (32–48) points. Only 15% of the participants reported normal fatigue, whilst almost 68% of the participants reported to experience severe fatigue, ranging from 46% in people with kidney disease to 95% in people with ME/CFS (Figure 1). In the subset of participants who reported not experiencing fatigue (*n* = 254, 6%), still 77 patients (31%) had CIS-Fatigue scores indicative of mild to severe fatigue.

**Figure 2.**
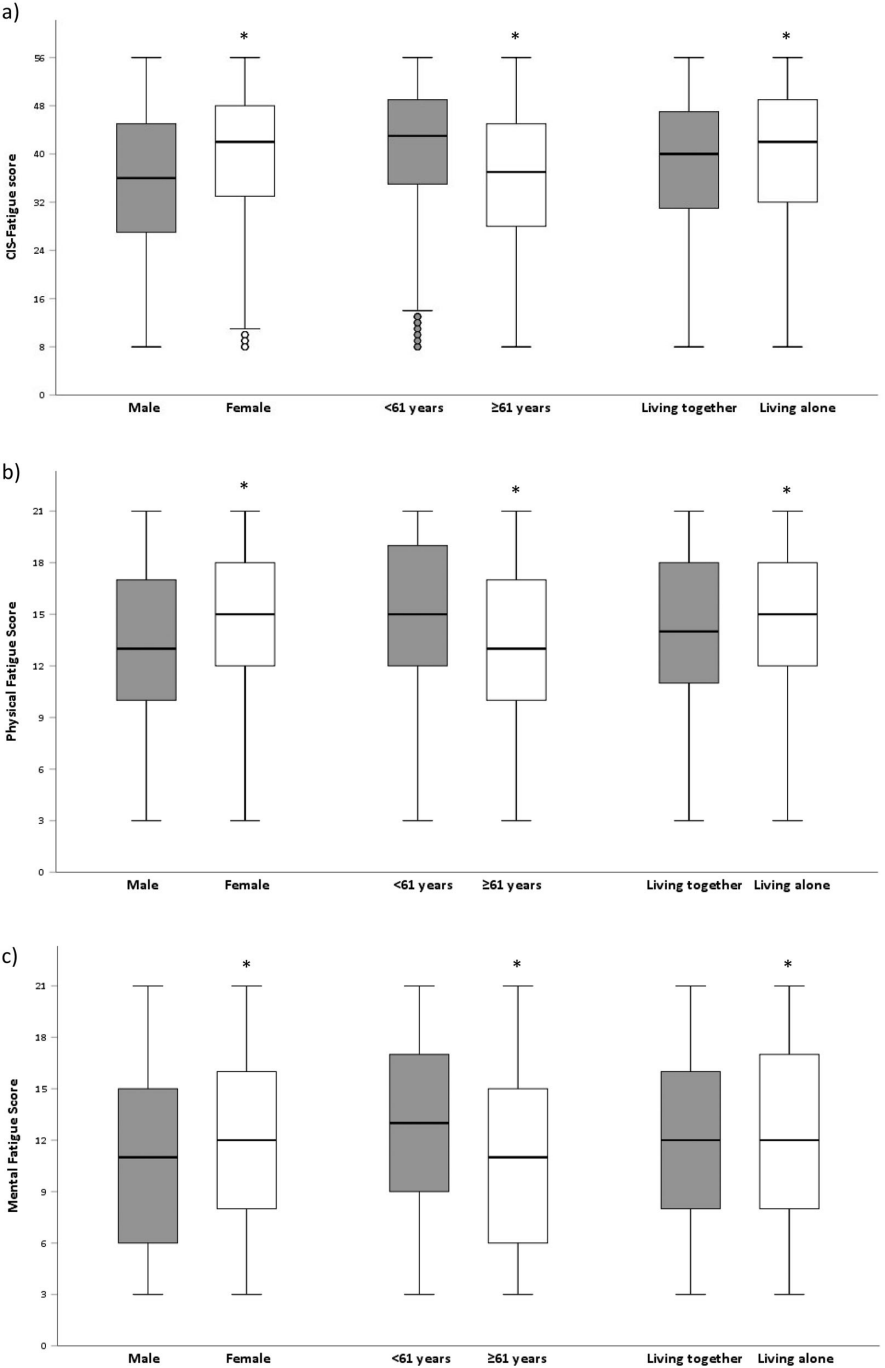
Fatigue stratified by sex, age and marital status; *p < .05; ^◦^outliers. a) CIS-Fatigue scores; b) Physical Fatigue Scores; c) Mental Fatigue scores.

Participants reported median physical and mental fatigue scores of 15 (11–18) points and 12 (8–16) points, respectively (Table 2). CIS-Fatigue scores and physical and mental fatigue scores were significantly higher in females, participants aged 60 or younger and participants living alone (Figure 2).

Physical and mental fatigue scores stratified by patient association are shown in Supplemental Figure 1. Significant differences were found between patient associations, with generally the highest median physical fatigue scores reported by members of the Dutch ME/CFS Foundation (19 points; *p* < .05 *vs.* members of other patients associations), and the highest median mental fatigue scores by participants with brain disorders, participants with mental health disorders and participants with ME/CFS (16 points; *p* < .05 *vs.* members of other patients associations).

**Figure 3.**
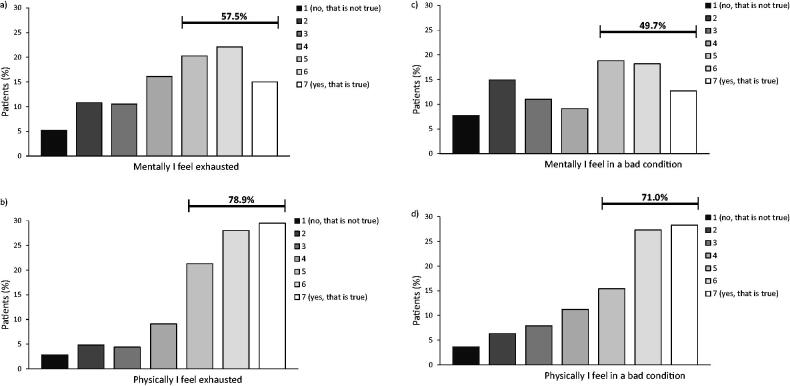
Physical and mental fatigue. a) Scores on question “Mentally I feel exhausted” in people feeling physically exhausted (*n* = 2221). b) Scores on question “Physically I feel exhausted” in people feeling mentally exhausted (*n* = 1619). c) Scores on question “Mentally I feel I am in a bad condition” in people feeling physically in a bad condition (*n* = 2165). d) Scores on question “Physically I feel I am in a bad condition” in people feeling mentally in a bad condition (*n* = 1515).

A total of 2221 participants (53%) felt physically exhausted, of which 58% also reported to feel mentally exhausted (Figure 3(a)). Of the 1619 participants (39%) feeling mentally exhausted, 79% also felt physically exhausted (Figure 3(b)).

A total of 2165 participants (52%) reported feeling physically in a bad condition. Of these, 50% also felt mentally in a bad condition (Figure 3(c)). Of the 1515 participants (36%) feeling mentally in a bad condition, 71% reported to also feeling physically in a bad condition (Figure 3(d)).

### Prescribed treatment for fatigue

**Figure 4.**
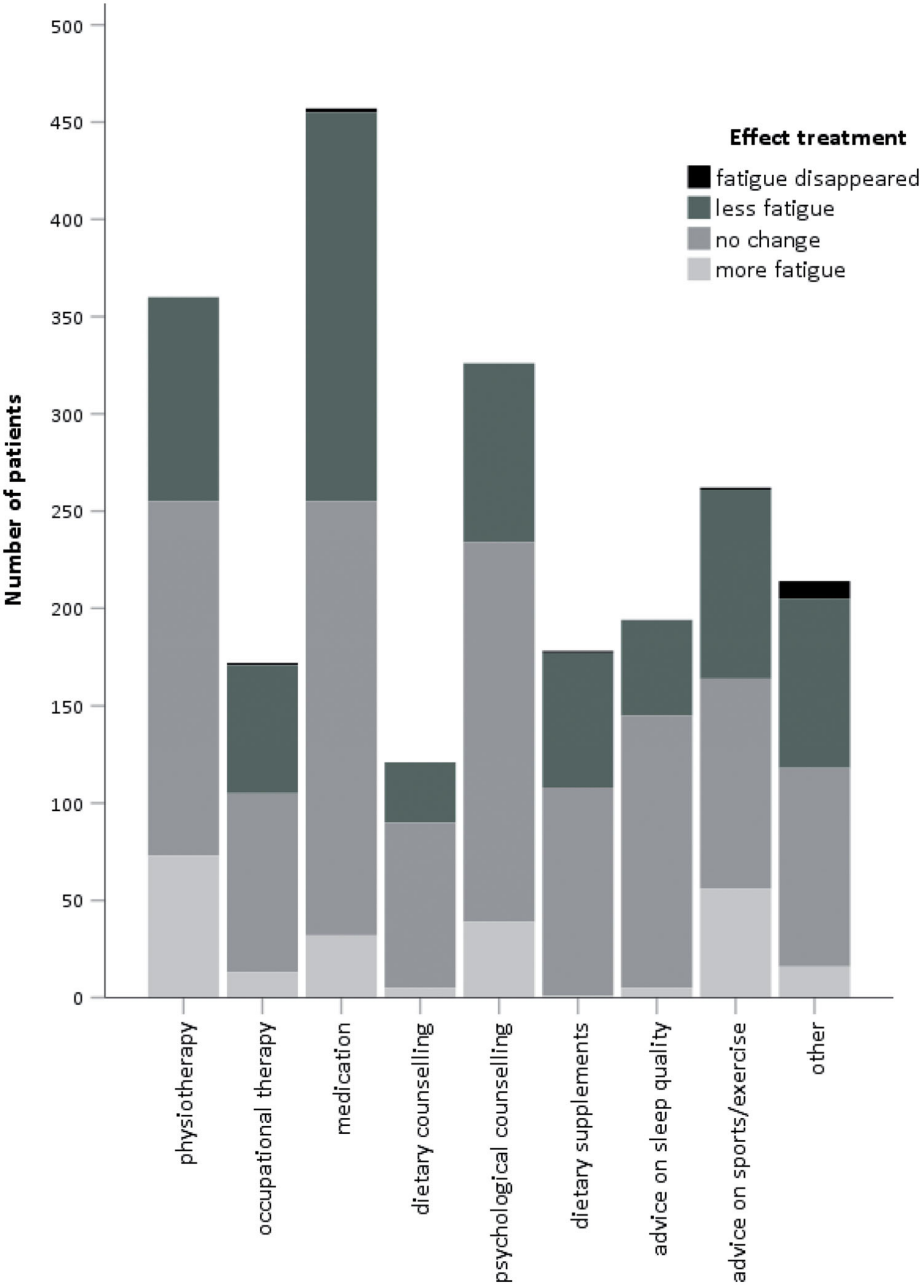
Prescribed treatment for fatigue in 896 participants.

Of 45% of the participants reported that they regularly or always discussed their fatigue during the consultation with their general practitioner or medical specialist, whilst 45% discussed it rarely or occasionally and 10% never discussed fatigue during the consultation. Less than a quarter of the participants (23%) reported being prescribed a treatment for fatigue, of which medication (51%), physiotherapy (40%) and psychological counselling or cognitive behavioural therapy (36%) was the most common (Figure 4). Most often reported other prescribed treatments were multidisciplinary rehabilitation program (25%), sleep apnoea treatment (15%) and vitamin B12 injections (7%).

Of the participants who regularly or always discussed their fatigue with a healthcare professional, the vast majority (65%) were not prescribed any treatment. In general, participants reported that treatment had no effect on their level of fatigue or felt this even resulted in an increase in fatigue (ranging from 56% for medication to 75% for advice on sleep; Figure 4). Prescribed treatment stratified by patient association is shown in Supplemental Figure 2.

## Discussion

This survey of 4199 participants with non-communicable chronic diseases which are member of patient associations, has the following important findings: 1) Severe fatigue is frequently (68%) present in people with non-communicable chronic diseases; 2) Physical and mental fatigue are often experienced simultaneously (64%), though these can also occur separately; 3) More than half of the participants never, rarely or only occasionally discussed fatigue with their healthcare professionals (55%); and 4) Only a small proportion of the people with non-communicable chronic diseases (23%) have been prescribed treatment for fatigue, which was often perceived as ineffective.

This study corroborates earlier findings that fatigue is commonly reported across different non-communicable chronic diseases [4,5,8,9]. Indeed, more than two-thirds of participants reported to experience severe fatigue, whilst only a small proportion of the participants reported normal fatigue levels. Where clinical research in non-communicable chronic diseases mainly focussed on the physical experience of fatigue (i.e. lack of energy and decreased physical performance) [9,14], we demonstrated that both physical and mental fatigue are common in participants with non-communicable chronic diseases. In line with previous studies, we demonstrated that fatigue scores were significantly higher in females, younger participants (below the age of 60) and participants living alone [4,5]. Interestingly, people with mental diseases also reported high levels of physical fatigue, whilst in the primarily physical diseases (e.g. participants with rheumatoid arthritis, neuromuscular diseases or burns) mental fatigue was also frequently present.

People with chronic diseases often consider fatigue as their most debilitating symptom, having a serious impact on daily functioning and quality of life [9]. Despite this, fatigue is often a neglected symptom in the management of chronic diseases. Because of the subjective nature of fatigue and insufficient knowledge of adequate treatment strategies for fatigue, it is often ignored or insufficiently evaluated by healthcare professionals [9]. This might, at least in part, be due to a limited understanding of the underlying causes of fatigue and the fact that fatigue-related questions are underrepresented in commonly used health status assessment tools [24]. Our data also demonstrated that less than half of the participants who reported to experience fatigue frequently discussed fatigue with their general practitioner or medical specialist. People often express feelings of misunderstanding by healthcare providers, but also by their loved ones, which can have a negative impact on their psychosocial well-being [9]. This emphasizes the need to create awareness among healthcare professionals for adequate screening and evaluating of fatigue in people with non-communicable chronic disease, which may even be more relevant in people with multi-morbidity, who have shown to have a higher risk of severe fatigue [5].

Our findings showed that people with non-communicable chronic diseases frequently experience physical fatigue and mental fatigue, either alone or a combination of both. This underlines the importance to take into account the different dimensions in the assessment of fatigue. More insight in the nature of fatigue may contribute to more optimal care.

Recently, it was suggested that several factors underlying fatigue were similar for multiple non-communicable chronic diseases, including female gender, younger age, higher BMI, being a current smoker, increased resting heart rate, reduced motivation, symptoms of anxiety and/or depression, pain, sleep disturbances, limitations in physical functioning and not being involved in leisure-time sports activities [4,5]. This provides a basis for using a trans-diagnostic approach for the management of fatigue in non-communicable chronic diseases rather than interventions tailored to specific chronic diseases [4,5,25]. To date, fatigue management interventions including cognitive behavioural therapy and/or exercise therapy appear to be the most promising across different diseases, for example rheumatoid-arthritis [26], COPD [27], type 1 diabetes mellitus [28], multiple sclerosis [29] and end-stage renal failure [30]. Then again, evidence on the effectiveness of cognitive behavioural therapy and/or exercise therapy for reducing fatigue in patients with ME/CFS is conflicting and the added value compared to usual care is relatively small [31–34], which may indicate that in these patients other mechanisms underlying fatigue may be present. Then again, cognitive behavioural therapy and/or exercise therapy might be effective in a specific subset of patients with ME/CFS. Of note, earlier articles investigating factors associated with fatigue across different chronic disease did not include patients with ME/CFS [4,5]. Interestingly, our findings also demonstrated that less than 30% of the participants with non-communicable chronic diseases reported an improvement in fatigue after exercise-based therapy or psychological counselling or cognitive behavioural therapy. Even more remarkably, only less than a quarter of the participants who reported to experience fatigue in this study had been prescribed a treatment for fatigue, and these were often perceived to be ineffective. This once again demonstrates the need to raise awareness of fatigue among healthcare professionals. Moreover, future studies are needed to develop effective evidence-based fatigue management interventions for people with non-communicable chronic diseases, taking into account the multifactorial nature of fatigue and differences in contributing pathophysiological mechanisms of fatigue. Furthermore, more insight in the pathophysiology of fatigue and contributing mechanisms will be relevant to identify candidates who are likely to benefit from specific fatigue management interventions.

### Methodological considerations

A clear strength of this study is the large amount of participants with different non-communicable chronic diseases. Moreover, this study focussed on both the physical and mental component of fatigue. A better understanding of the experience of fatigue in multiple non-communicable chronic conditions may be the first step for an improved management of this symptom.

This study has the following limitations: 1) There is the possibility of selection bias, as it is reasonable to assume that participants experiencing fatigue are more likely to complete the questionnaire. Therefore, it is possible that the true prevalence of fatigue in this study was overestimated. 2) Participants were all members of patient associations, though we did not have objective information about the actual diagnoses or the presence of possible comorbidities. Moreover, the degree of disease severity remains unknown. Then again, disease markers of the primary chronic organ failure are not or only poorly related to the degree of fatigue [6,35–38]. 3) Participants were mostly females, and the proportion of older people was relatively low, which limits the generalizability of our findings. Moreover, the prevalence of ethnic minority groups was low, which may at least partly be explained by the fact that participants who could not understand the Dutch language were unable to complete the questionnaire. 4) Almost half of the participants were member of the Dutch Arthritis Society. Furthermore, participants from some patient associations were under represented. Therefore, this skewed distribution of participants from different patient associations may limit the generalizability of the current findings. 5) The inclusion of patients with ME/CFS may have biased the results, as it can be expected that all patients with ME/CFS experience severe fatigue. However, sensitivity analysis excluding these patients yielded similar findings for prevalence and severity of fatigue. 6) The assessment of fatigue may be subject to recall bias and does not capture diurnal variation of fatigue. Future studies may want to use methods, such as ecological momentary assessment to overcome this limitation [39–41]. 7) Although validated questionnaires (such as the Chalder fatigue index) to assess mental (and physical) fatigue are available [42], self-constructed questions were used to quantify mental fatigue in this study, as was done before in patients with long-COVID [20]. Moreover, no validated cut-offs were available to classify individuals as having normal/mild/severe physical or mental fatigue. Nevertheless, our findings indicate that participants with non-communicable chronic diseases experience both physical and mental fatigue. 8) Specific information on prescribed treatment is lacking, such as type of medication or physiotherapeutic modalities (endurance and strength). Future studies are needed to further explore the effectiveness of specific fatigue management interventions for people with non-communicable chronic diseases.

## Conclusion

Our findings indicate that both physical and mental fatigue are often experienced simultaneously in non-communicable chronic diseases, but can also occur separately. More than half of the participants never, rarely or only occasionally discussed fatigue with their healthcare professionals. So, healthcare professionals need to be aware of the high prevalence of fatigue in non-communicable chronic diseases, and the need for regular assessment of the presence and severity of fatigue. Furthermore, in only less than a quarter of the people with non-communicable chronic diseases treatment for fatigue was prescribed, which often was experienced as ineffective. Future studies are needed to gain more insight in underlying factors of fatigue in non-communicable chronic diseases, its impact on daily life and possible treatment strategies.

## Supplementary Material

Supplemental Material

Supplemental Material

Supplemental Material

## Data Availability

The data that support the findings of this study are available from the corresponding author upon reasonable request.

## Appendix 1. Online questionnaire

1. What is your sex?
    ☐ Male
    ☐ Female
    ☐ Other/I do not want to say
2. What is your age?
    ☐ 18–30 years
    ☐ 31–40 years
    ☐ 41–50 years
    ☐ 51–60 years
    ☐ 61–70 years
    ☐ 71–80 years
    ☐ 81 years and older
3. What is your ethnicity?
    ☐ White
    ☐ Hispanic/Latino
    ☐ Black/African American
    ☐ Asian/Pacific Islander
    ☐ Other/I do not want to say
4. What is your marital status?
    ☐ Living alone
    ☐ Married/living together
    ☐ Divorced
    ☐ Widow/widower
5. Do you have children?
    ☐ No
    ☐ Yes, children living at home……………….. (number)
    In the age group:
       ☐ 0–5 years
       ☐ 6–10 years
       ☐ 11–15 years
       ☐ 16–20 years
       ☐ 21 years and older
    ☐ Yes, children living away from home………… ……..(number)
6. What is your highest completed education?
    ☐ No education/incomplete primary education
    ☐ Primary education
    ☐ Lower vocational education/pre-vocational education (e.g. LTS and VMBO)
    ☐ General secondary education (e.g. MAVO, MULO, ULO, MBO-kort and VMBO-t)
    ☐ Secondary vocational education (e.g. MBO, MTS, MEAO, BOL and BBL)
    ☐ Senior general secondary education/pre-university education (e.g. HAVO, VWO, Athenaeum, Gymnasium and HBS)
    ☐ Higher professional education (e.g. HBO, HTS, HEAO, HBO-V and PABO)
    ☐ Academic higher education (university) or higher
7. Do you currently have paid employment, and if so, for how many hours?
    ☐ No
    ☐ Yes, full-time (36 h/week or more)
    ☐ Yes, part-time: ……… hours/week
8. You have received this questionnaire *via*:
    ☐ Lung Foundation Netherlands
    ☐ Dutch Heart Foundation
    ☐ Dutch Kidney Foundation
    ☐ Dutch Diabetes Foundation
    ☐ Princess Beatrix Muscle Foundation
    ☐ Dutch Neuromuscular Disease Association
    ☐ Dutch Arthritis Society
    ☐ Dutch Digestive Disease Foundation
    ☐ Dutch Brain Foundation
    ☐ Dutch Foundation for Mental Health
    ☐ Dutch Burn Foundation
    ☐ Irritable Bowel Syndrome Patient Association
    ☐ Dutch ME/CFS Foundation
    ☐ Dutch Patient Association for Cardiovascular Diseases
    ☐ Other:……………………………… ………………………………
9. Do you now experience or have you ever had complaints of fatigue?
    ☐ Yes
    ☐ No -> jump to question 16
10. Which applies most to you?
    ☐ Fatigue is/was mostly physical fatigue
    ☐ Fatigue is/was mostly mental fatigue
    ☐ Fatigue is/was a combination of physical and mental fatigue
11. How often have you discussed your fatigue with your doctor during a consultation?
    ☐ Never
    ☐ Once
    ☐ Rarely
    ☐ Occasionally
    ☐ Regularly
    ☐ Every time
12. Have you had any doctor prescribed treatment for fatigue in the past 5 years?
    ☐ Yes
    ☐ No -> jump to question 16
13. What did this prescribed treatment for fatigue consist of? (You may select multiple answers)
    ☐ Physiotherapy
    ☐ Occupational therapy
    ☐ Medication
    ☐ Dietary counselling
    ☐ Psychological counselling/cognitive behavioural therapy
    ☐ Dietary supplements
    ☐ Advice on sleep quality
    ☐ Advice on sports/exercise
    ☐ Other: ………………………………… ……………………………… ….………………………………….
14. Did the prescribed treatment affect your fatigue?
  After the [mentioned treatment(s) at question 13] I experienced:
    ☐ No fatigue
    ☐ Less fatigue
    ☐ No change in fatigue -> jump to question 16
    ☐ More fatigue
15. Was there a temporary change or a long-term change?
    ☐ Temporary change
    ☐ Long-term change

**Checklist individual strength subscale subjective fatigue**

Below you will find 11 statements with which you can indicate how you have been feeling during the past two weeks.

You can answer each question by circle one of the seven numbers. The number indicates to what extent you think the statement applies to you.

For example, if you think it is completely true, circle the left number, so like this:

If you think the answer is neither “yes, that is true”, nor “no, that is not true”, circle the number that most closely matches your feeling. For example like this:

Answer all eleven statements and circle *one number for each statement*.

**Table:**

1.	I feel tired.	Yes, that is true	1	2	3	4	5	6	7	No, that is not true
										
2.	Physically I feel exhausted.	Yes, that is true	1	2	3	4	5	6	7	No, that is not true
										
3.	I feel fit.	Yes, that is true	1	2	3	4	5	6	7	No, that is not true
										
4.	I feel weak.	Yes, that is true	1	2	3	4	5	6	7	No, that is not true
										
5.	I feel rested.	Yes, that is true	1	2	3	4	5	6	7	No, that is not true
										
6.	Physically I feel I am in a bad condition.	Yes, that is true	1	2	3	4	5	6	7	No, that is not true
										
7.	I get tired very quickly.	Yes, that is true	1	2	3	4	5	6	7	No, that is not true
										
8.	Physically I feel in a good shape.	Yes, that is true	1	2	3	4	5	6	7	No, that is not true
										
9.	Mentally I feel exhausted.	Yes, that is true	1	2	3	4	5	6	7	No, that is not true
										
10.	Mentally I feel I am in a bad condition.	Yes, that is true	1	2	3	4	5	6	7	No, that is not true
										
11.	Mentally I feel in a good shape.	Yes, that is true	1	2	3	4	5	6	7	No, that is not true

**Scoring**

For the items: 3, 5, 8, 11 the scoring is as follows:

**Table:**

Yes, that is true	1	2	3	4	5	6	7	No, that is not true

For the items: 1, 2, 4, 6, 7, 9, 10 the scoring is as follows:

**Table:**

Yes, that is true	7	6	5	4	3	2	1	No, that is not true
