# Supplementary material for: Physical and mental fatigue in people with non-communicable chronic diseases
Source: Ann Med. 2022 Sep 16;54(1):2522–34. doi: 10.1080/07853890.2022.2122553 (PMC9487929; doi:10.1080/07853890.2022.2122553)
Supplement: Supplemental Material [file IANN_A_2122553_SM4403.docx]

**Supplementary table 1** Patient characteristics and fatigue-related outcomes stratified by patient association

|  | **Dutch Heart Foundation (Hartstichting)** | **Dutch Arthritis Society (ReumaNederland)** | **Lung Foundation Netherlands (Longfonds)** | **Dutch Kidney Foundation (Nierstichting** | **Dutch Diabetes Foundation (Diabetes Fonds)** | **Princess Beatrix Muscle Foundation (Prinses Beatrix Spierfonds)** | **Dutch Neuromuscular Disease Association (Spierziekten Nederland)** | **Dutch Digestive Disease Foundation (Maag Lever Darm Stichting)** | **Dutch Brain Foundation (Hersenstichting)** | **Dutch Foundation for Mental Health (MIND)** | **Dutch Burn Foundation (Brandwonden Stichting)** | **Irritable Bowel Syndrom Patient Association (Prikkelbare Darm Syndroom Belangenorganisatie)** | **Dutch ME/CFS Foundation (ME/CVS Stichting)** | **Dutch Patient Association for Cardiovascular Diseases (Harteraad)** |
| --- | --- | --- | --- | --- | --- | --- | --- | --- | --- | --- | --- | --- | --- | --- |
| **N (%)** | 544 (13.0) | 2060 (49.1) | 167 (4.0) | 159 (3.8) | 73 (1.7) | 58 (1.4) | 213 (5.1) | 337 (8.0) | 28 (0.7) | 43 (1.0) | 103 (2.5) | 22 (0.5) | 250 (6.0) | 142 (3.4) |
| **Sex, n (%)**  Male  Female  Other/I don’t want to say | 119 (21.9)  421 (77.4)  4 (0.7) | 394 (19.1)  1663 (80.7)  3 (0.1) | 59 (35.3)  107 (63.0)  1 (0.6) | 93 (58.5)  66 (41.5)  - | 21 (28.8)  52 (71.2)  - | 8 (13.8)  50 (86.2)  - | 73 (34.3)  140 (65.7)  - | 29 (8.6)  308 (91.4)  - | 5 (17.9)  23 (82.1)  - | 7 (16.3)  36 (83.7  -) | 35 (34.0)  68 (66.0)  - | 2 (9.1)  20 (90.9)  - | 41 (16.4)  208 (83.2)  1 (0.4) | 65 (45.8)  77 (54.2)  - |
| **Age, years, n (%)**  18-30  31-40  41-50  51-60  61-70  71-80  ≥81 | 16 (2.9)  29 (5.3)  101 (18.6)  199 (36.6)  137 (25.2)  57 (10.5)  5 (0.9) | 36 (1.7)  93 (4.5)  265 (12.9)  545 (26.5)  730 (35.4)  361 (17.5)  30 (1.5) | 6 (3.6)  9 (5.4)  21 (12.6)  36 (21.6)  65 (38.9)  26 (15.6)  4 (2.4) | 4 (2.5)  9 (5.7)  10 (6.3)  36 (22.6)  68 (42.8)  28 (17.6)  4 (2.5) | 24 (32.9)  14 (19.2)  13 (17.8)  10 (13.7)  10 (13.7)  2 (2.7)  - | 10 (17.2)  11 (19.0)  12 (20.7)  13 (22.4)  11 (19.0)  1 (1.7)  - | 12 (5.6)  16 (7.5)  29 (13.6)  60 (28.2)  54 (25.4)  38 (17.8)  4 (1.9) | 67 (19.9)  73 (21.7)  92 (27.3)  72 (21.4)  26 (7.7)  7 (2.1)  - | 1 (3.6)  2 (7.1)  10 (35.7)  9 (32.1)  6 (21.4)  -  - | 9 (20.9)  13 (30.2)  8 (18.6)  9 (20.9)  3 (7.0)  1 (2.3)  - | 12 (11.7)  21 (20.4)  26 (25.2)  16 (15.5)  23 (22.3)  5 (4.9)  - | 6 (27.3)  2 (9.1)  4 (18.2)  8 (36.4)  1 (4.5)  1 (4.5)  - | 36 (14.4)  40 (16.0)  44 (17.6)  77 (30.8)  41 (16.4)  10 (4.0)  2 (0.8) | 2 (1.4)  7 (4.9)  18 (12.7)  41 (28.9)  46 (32.4)  26 (18.3)  2 (1.4) |
| **Ethnicity, n (%)**  White  Hispanic/Latino  Black/African American  Asian/ Pacifc Islander  Other/ I don’t want to say | 533 (98.0)  1 (0.2)  1 (0.2)  4 (0.7)  5 (0.9) | 1994 (96.8)  7 (0.3)  5 (0.2)  32 (1.6)  22 (1.1) | 162 (97.0)  1 (0.6)  -  2 (1.2)  2 (1.2) | 158 (99.4)  -  -  1 (0.6)  - | 68 (93.2)  -  -  4 (5.5)  1 (1.4) | 58 (100.0)  -  -  -  - | 210 (98.6)  -  -  1 (0.5)  2 (0.9) | 324 (96.1)  -  2 (0.6)  5 (1.5)  6 (1.8) | 26 (92.9)  -  -  1 (3.6)  1 (3.6) | 42 (97.7)  -  -  -  1 (2.3) | 93 (90.3)  -  4 (3.9)  2 (1.9)  4 (3.9) | 21 (95.5)  -  -  1 (4.5)  - | 241 (96.4)  1 (0.4)  -  2 (0.8)  6 (2.4) | 135 (95.1)  -  -  5 (3.5)  2 (1.4) |
| **Marital status, n (%)**  Living alone  Married/Living together  Divorced  Widow/widower | 83 (15.3)  412 (75.7)  32 (5.9)  17 (3.1) | 379 (18.4)  1414 (68.6)  138 (6.7)  129 (6.3) | 33 (19.8)  123 (73.3)  9 (5.4)  2 (1.2) | 28 (17.6)  122 (76.7)  6 (3.8)  3 (1.9) | 21 (28.8)  49 (67.1)  3 (4.1)  - | 20 (34.5)  35 (60.3)  2 (3.4)  1 (1.7) | 35 (16.4)  160 (75.1)  11 (5.2)  7 (3.3) | 78 (23.1)  240 (71.2)  17 (5.0)  2 (0.6) | 5 (17.9)  22 (78.6)  1 (3.6)  - | 17 (39.5)  21 (48.8)  3 (7.0)  2 (4.7) | 29 (28.2)  62 (60.2)  10 (9.7)  2 (1.9) | 3 (13.6)  15 (68.2)  3 (13.6)  1 (4.5) | 79 (31.6)  154 (61.6)  15 (6.0)  2 (0.8) | 22 (15.5)  108 (76.1)  7 (4.9)  5 (3.5) |
| **Children, n (%)**  Yes  *Children living at home*  No | 423 (77.8)  280 (66.2)  121 (22.2) | 1415 (68.7)  687 (48.6)  645 (31.3) | 107 (64.1)  58 (54.2)  60 (35.9) | 85 (53.5)  45 (52.9)  74 (46.5) | 35 (47.9)  24 (68.6)  38 (52.1) | 30 (51.7)  18 (60.0)  28 (48.3) | 142 (66.7)  68 (47.9)  71 (33.3) | 204 (60.5)  155 (76.0)  133 (39.5) | 17 (60.7)  10 (58.8)  11 (39.3) | 19 (44.2)  15 (78.9)  24 (55.8) | 64 (62.1)  50 (78.1)  39 (37.9) | 13 (59.1)  10 (76.9)  9 (40.9) | 114 (45.6)  80 (70.2)  136 (54.5) | 101 (71.1)  55 (54.5)  41 (28.9) |
| **Education level, n (%)**  Low  Moderate  High | 151 (27.8)  217 (39.9)  176 (32.4) | 461 (22.4)  763 (37.0)  836 (40.6) | 32 (19.2)  52 (31.1)  83 (49.7) | 29 (18.2)  44 (27.7)  86 (54.1) | 7 (9.6)  29 (39.7)  37 (50.7) | 13 (67.2)  27 (46.6)  18 (31.0) | 42 (19.7)  72 (33.8)  99 (46.5) | 47 (13.9)  141 (41.8)  149 (44.2) | 1 (3.6)  16 (57.1)  11 (39.5) | 5 (14.0)  8 (18.6)  29 (67.4) | 22 (21.4)  44 (42.7)  37 (35.9) | 5 (22.7)  12 (54.5)  5 (22.7) | 42 (16.8)  102 (40.8)  106 (42.4) | 28 (19.7)  48 (33.8)  66 (46.5) |
| **Work situation, n (%)**  Full-time job  Part-time job  No paid employment | 75 (13.8)  169 (31.1)  300 (55.1) | 174 (8.4)  521 (25.3)  1365 (66.3) | 13 (7.8)  40 (24.0)  114 (68.3) | 14 (8.8)  27 (17.0)  118 (74.2) | 19 (26.0)  30 (41.1)  24 (32.9) | 4 (6.9)  15 (25.9)  39 (67.2) | 14 (6.6)  57 (26.8)  142 (66.7) | 68 (20.2)  144 (42.7)  125 (37.1) | 1 (3.6)  10 (35.7)  17 (60.7) | 3 (7.0)  18 (41.9)  22 (51.2) | 23 (22.3)  39 (37.9)  41 (39.8) | 4 (18.2)  7 (31.8)  11 (50.0) | 4 (1.6)  62 (24.8)  184 (73.6) | 19 (13.4)  27 (19.0)  96 (67.6) |

| **Experiencing fatigue, n(%)**  Mainly physical fatigue  Mainly mental fatigue  Both physical and mental fatigue | 520 (95.6)  178 (34.2)  14 (2.7)  328 (63.1) | 1908 (92.6)  618 (32.4)  39 (2.0)  1251 (65.6) | 162 (97.0)  72 (44.4)  5 (3.1)  85 (52.5) | 145 (91.2)  66 (45.5)  4 (2.8)  75 (51.7) | 69 (94.5)  18 (26.1)  5 (7.2)  46 (66.7) | 56 (96.6)  19 (33.9)  1 (1.8)  36 (64.3) | 202 (94.8)  80 (39.6)  5 (2.5)  117 (57.9) | 330 (97.9)  109 (33.0)  10 (3.0)  211 (63.9) | 27 (96.4)  4 (14.8)  1 (3.7)  22 (81.4) | 41 (95.3)  2 (4.9)  10 (24.4)  29 (70.7) | 86 (83.5)  18 (20.9)  6 (7.0)  62 (72.1) | 20 (90.9)  4 (20.0)  1 (5.0)  15 (75.0) | 249 (99.6)  63 (25.3)  1 (0.4)  185 (74.3) | 130 (91.5)  48 (36.9)  4 (3.1)  78 (60.0) |
| --- | --- | --- | --- | --- | --- | --- | --- | --- | --- | --- | --- | --- | --- | --- |
| **General fatigue (CIS-F), median (IQR)** | 40 (32-47) | 39 (30-46) | 40 (32-46) | 33 (25-42) | 43 (33-48) | 42 (36-48) | 42 (35-49) | 39 (30-46) | 43 (37-49) | 42 (34-46) | 38 (27-46) | 43 (31-50) | 51 (46-54) | 40 (32-48) |
| **Severe fatigue, n (%)** | 372 (68.4) | 1292 (62.7) | 118 (70.7) | 73 (45.9) | 55 (75.3) | 45 (77.6) | 163 (76.5) | 276 (81.9) | 23 (82.1) | 32 (74.4) | 58 (56.3) | 15 (68.2) | 237 (94.8) | 94 (66.2) |
| **Physical fatigue, median (IQR)** | 15 (11-18) | 14 (10-17) | 15 (12-18) | 13 (9-16) | 15 (12-18) | 15 (12-18) | 15 (12-18) | 14 (10-17) | 16 (13-19) | 15 (12-17) | 13 (9-17) | 13 (11-20) | 19 (16-21) | 15 (11-18) |
| **Mental fatigue, median (IQR)** | 12 (9-16) | 12 (7-15) | 11 (8-14) | 10 (6-13) | 15 (9-18) | 12 (8-15) | 12 (7-16) | 12 (7-15) | 16 (11-19) | 16 (14-19) | 12 (8-17) | 14 (11-19) | 16 (12-19) | 12 (8-15) |
| **Discussed with healthcare professional**  Never  Once  Rarely  Occasionally  Regularly  Every time | 61 (11.7)  19 (3.7)  59 (11.3)  132 (25.4)  177 (34.0)  72 (13.8) | 194 10.2)  58 (3.0)  299 (15.7)  587 (30.8)  600 (31.4)  170 (8.9) | 20 (12.3)  12 (7.4)  20 (12.3)  24 (25.3)  61 (37.7)  8 (4.9) | 13 (9.0)  5 (3.4)  24 (16.6)  49 (33.8)  47 (32.4)  7 (4.8) | 18 (26.1)  2 (2.9)  12 (17.4)  19 (27.5)  12 (17.4)  6 (8.7) | 5 (8.9)  1 (1.8)  5 (8.9)  17 (30.4)  19 (33.9)  9 (16.1) | 17 (8.4)  10 (5.0)  23 (11.4)  51 (25.2)  74 (36.6)  27 (13.4) | 22 (6.7)  11 (3.3)  34 (10.3)  83 (25.2)  120 (36.4)  60 (18.2) | 2 (7.4)  3 (11.1)  2 (7.4)  7 (25.9)  11 (40.7)  2 (7.4) | 9 (22.0)  3 (7.3)  7 (17.1)  14 (34.1)  8 (19.5)  - | 19 (22.1)  7 (8.1)  14 (16.3)  28 (32.6)  14 (16.3)  4 (4.7) | 1 (5.0)  -  3 (15.0)  7 (35.0)  8 (40.0)  1 (5.0) | 4 (1.6)  4 (1.6)  8 (3.2)  48 (19.3)  137 (55.0)  48 (19.3) | 7 (5.4)  4 (3.1)  6 (4.6)  40 (30.8)  57 (43.8)  16 (12.3) |
| **Prescribed treatment**  Physiotherapy  Occupational therapy  Medication  Dietary counselling  Psychological counseling/cognitive behavioral therapy  Dietary supplements  Advice on sleep quality  Advice on sports/exercise  Other | 40 (7.7)  26 (5.0)  70 (13.5)  15 (2.9)  41 (7.9)  17 (3.3)  18 (3.5)  43 (8.3)  36 (6.9) | 149 (7.8)  62 (3.2)  187 (9.8)  31 (1.6)  105 (5.5)  51 (2.7)  79 (4.1)  87 (4.6)  72 (3.8) | 17 (10.5)  3 (1.9)  17 (10.5)  11 (6.8)  9 (5.6)  3 (1.9)  6 (3.6)  14 (8.6)  10 (6.2) | 3 (2.1)  -  11 (7.6)  5 (3.4)  3 (2.1)  2 (1.4)  1 (0.7)  5 (3.4)  9 (6.2) | -  -  1 (1.4)  1 (1.4)  5 (7.2)  2 (2.9)  1 (1.4)  2 (2.9)  - | 8 (14.3)  4 (7.1)  6 (10.7)  2 (3.6)  4 (7.1)  1 (1.8)  -  4 (7.1)  4 (7.1) | 26 (12.9)  21 (10.4)  21 (10.4)  5 (2.5)  24 (11.9)  14 (6.9)  11 (5.4)  17 (8.4)  11 (5.4) | 22 (6.7)  9 (2.7)  33 (10.0)  12 (3.6)  29 (8.8)  17 (5.2)  15 (4.5)  24 (7.3)  13 (4.2) | 2 (7.4)  3 (11.1)  2 (7.4)  2 (7.4)  5 (18.5)  2 (7.4)  2 (7.4)  3 (11.1)  3 (11.1) | 4 (9.8)  1 (2.4)  5 (12.2)  1 (2.4)  10 (24.4)  1 (2.4)  5 (12.2)  3 (7.3)  1 (2.4) | 7 (8.1)  2 (2.3)  10 (11.6)  1 (1.2)  6 (7.0)  2 (2.3)  4 (4.7)  4 (4.7)  5 (5.8) | 2 (10.0)  -  3 (15.0)  2 (10.0)  1 (5.0)  1 (5.0)  1 (5.0)  1 (5.0)  - | 70 (28.1)  39 (15.7)  70 (28.1)  30 (12.0)  79 (31.7)  59 (23.7)  41 (16.5)  45 (18.1)  40 (16.1) | 10 (7.7)  2 (1.5)  21 (16.2)  3 (2.3)  5 (3.8)  6 (4.6)  10 (7.7)  10 (7.7)  10 (7.7) |
