# Supplementary material for: Physical and mental fatigue in people with non-communicable chronic diseases
Source: Ann Med. 2022 Sep 16;54(1):2522–34. doi: 10.1080/07853890.2022.2122553 (PMC9487929; doi:10.1080/07853890.2022.2122553)
Supplement: Supplemental Material [file IANN_A_2122553_SM4402.docx]

**Supplemental Figure 2**

Prescribed treatment for fatigue stratified by patient association

1. Dutch Heart Foundation (Hartstichting) (n=127)
2. Dutch Arthritis Society (ReumaNederland) (n=338)
3. Lung Foundation Netherlands (Longfonds) (n=38)
4. Dutch Kidney Foundation (Nierstichting) (n=25)
5. Dutch Diabetes Foundation (Diabetes Fonds) (n=9)
6. Princess Beatrix Muscle Foundation (Prinses Beatrix Spierfonds) (n=16)
7. Dutch Neuromuscular Disease Association (Spierziekten Nederland) (n=55)
8. Dutch Digestive Disease Foundation (Maag Lever Darm Stichting) (n=72)
9. Dutch Brain Foundation (Hersenstichting) (n=10)
10. Dutch Foundation for Mental Health (MIND) (n=11)
11. Dutch Burn Foundation (Brandwonden Stichting) (n=20)
12. Dutch ME/CFS Foundation (ME/CVS Stichting) (n=137)
13. Dutch Patient Association for Cardiovascular Diseases (Harteraad) (n=35)
14. Irritable Bowel Syndrom Patient Association (Prikkelbare Darm Syndroom Belangenorganisatie) (n=3)

| a) | Dutch Heart Foundation (Hartstichting) (n=127)  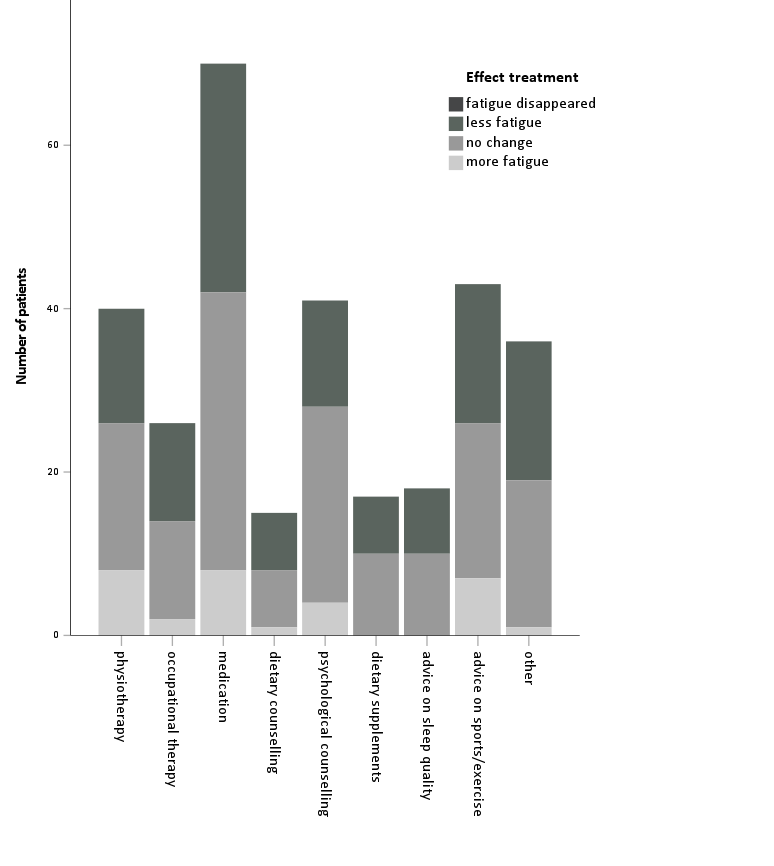 | b) | Dutch Arthritis Society (ReumaNederland) (n=338)  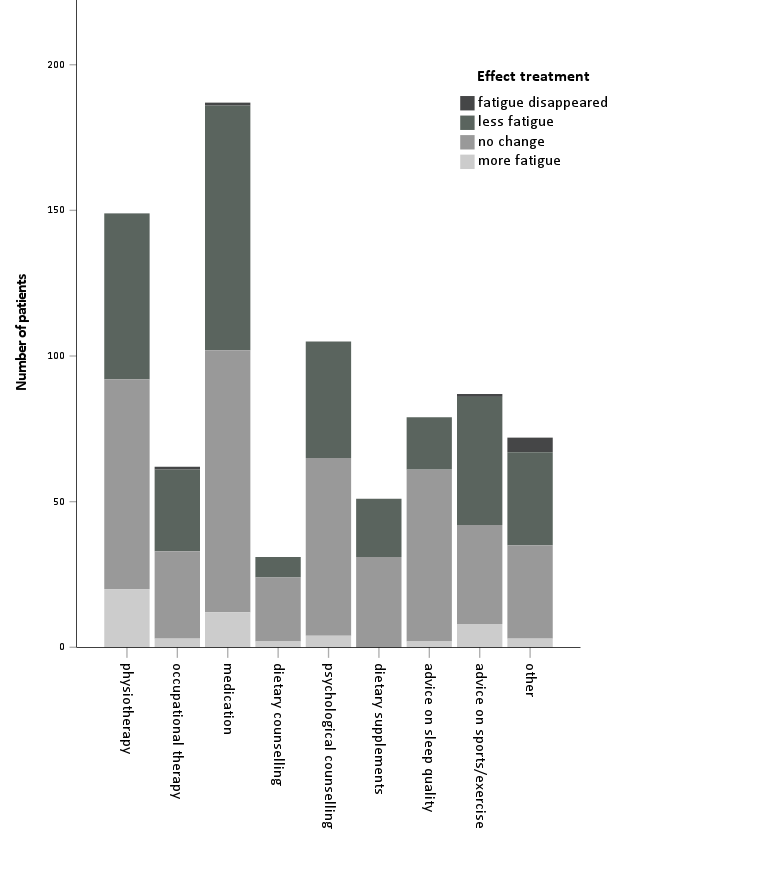 |
| --- | --- | --- | --- |

| c) | Lung Foundation Netherlands (Longfonds) (n=38)  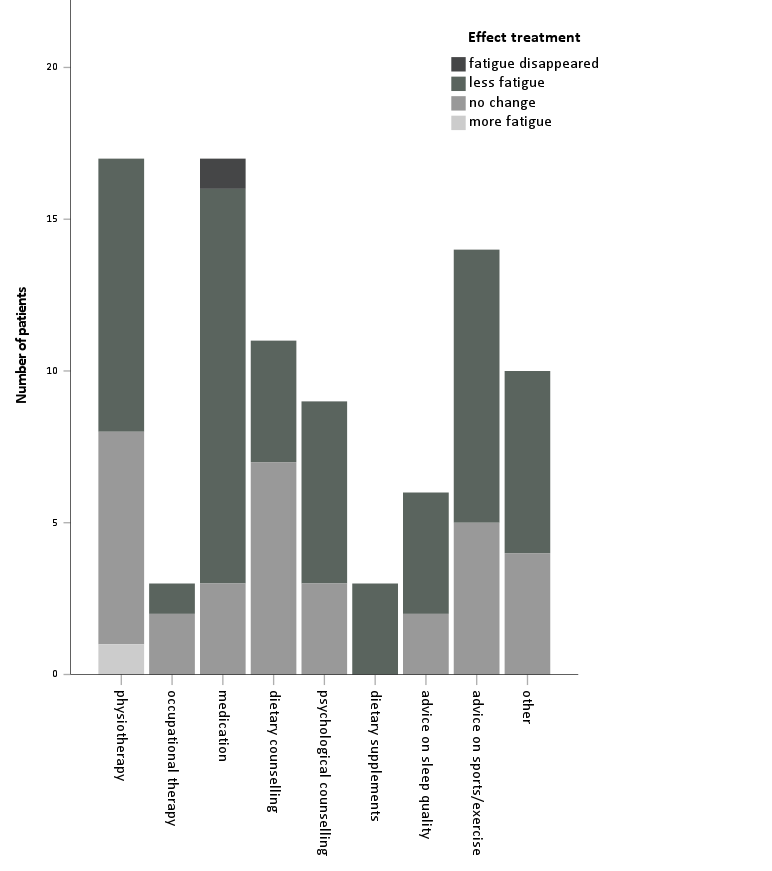 | d) | Dutch Kidney Foundation (Nierstichting) (n=25)  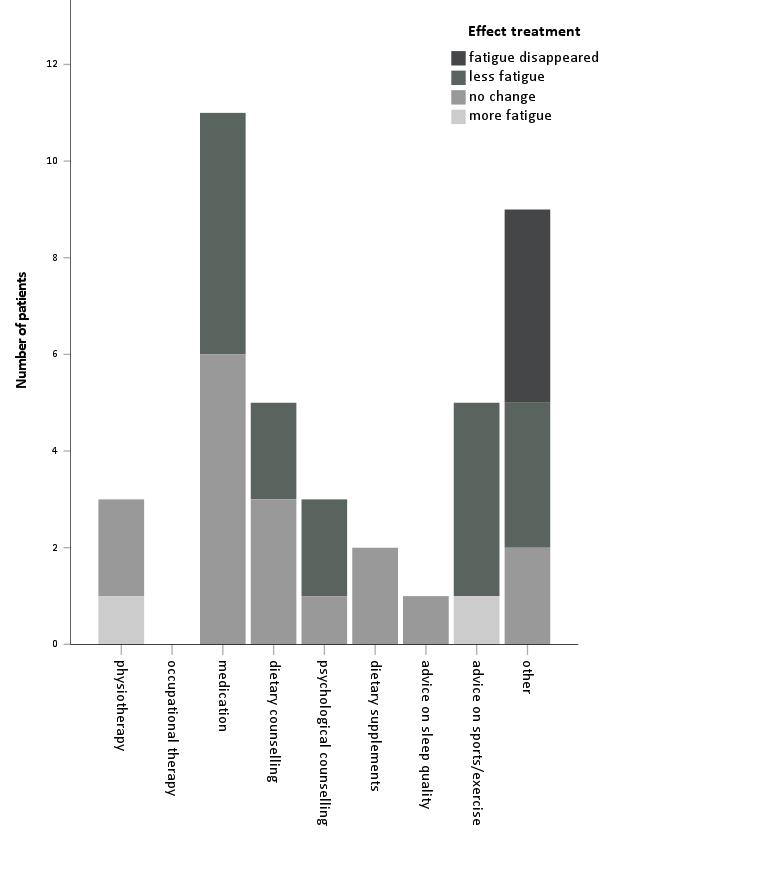 |
| --- | --- | --- | --- |

| e) | Dutch Diabetes Foundation (Diabetes Fonds) (n=9)  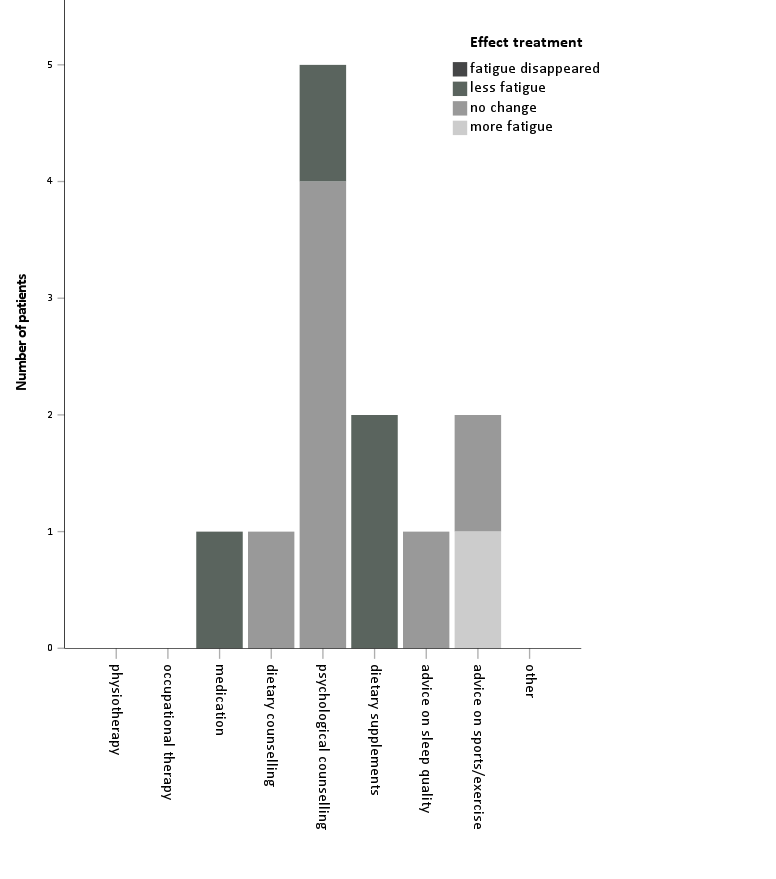 | f) | Princess Beatrix Muscle Foundation (Prinses Beatrix Spierfonds) (n=16)  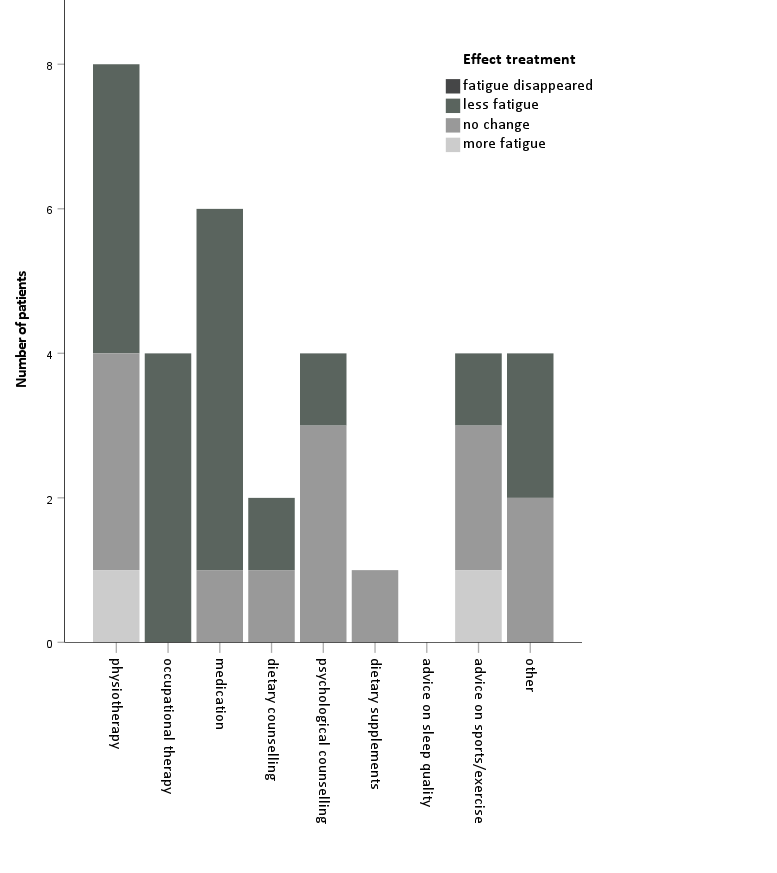 |
| --- | --- | --- | --- |

| g) | Dutch Neuromuscular Disease Association (Spierziekten Nederland) (n=55)  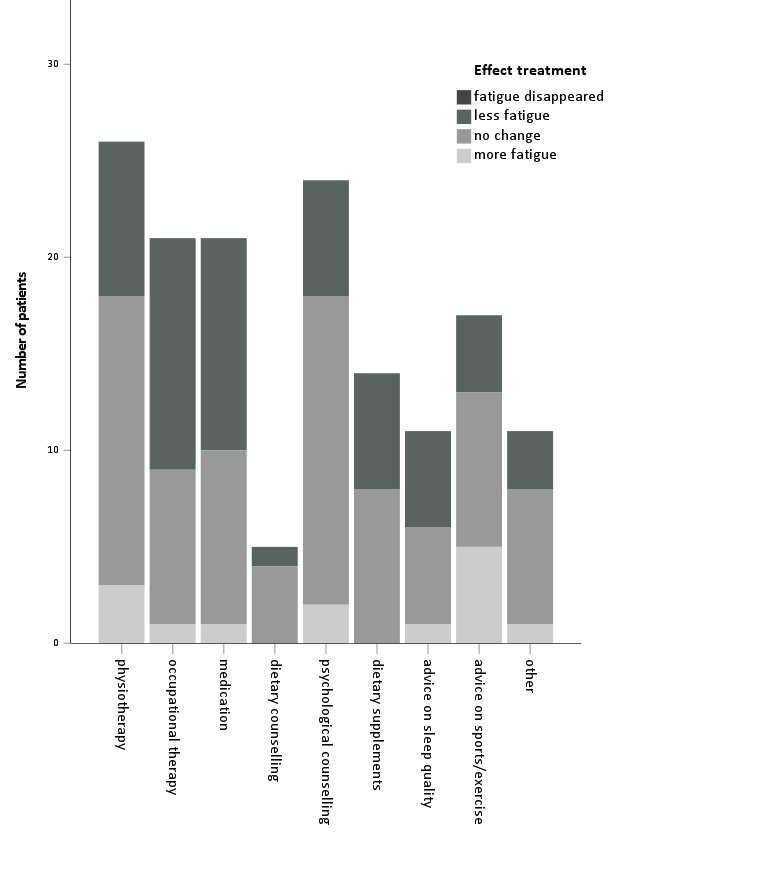 | h) | Dutch Digestive Disease Foundation (Maag Lever Darm Stichting) (n=72)  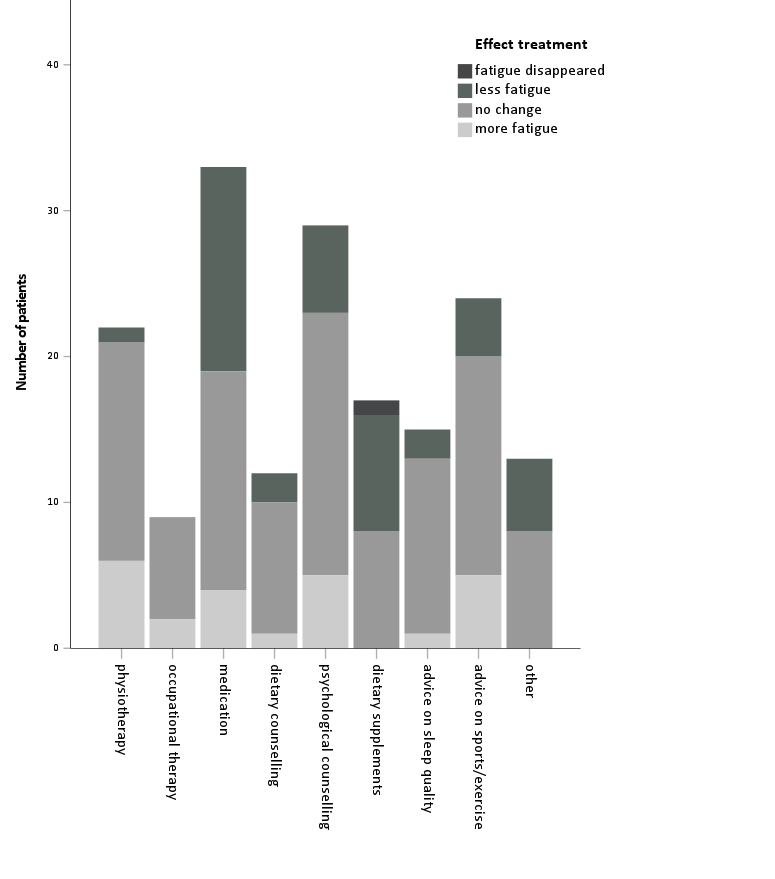 |
| --- | --- | --- | --- |

| i) | Dutch Brain Foundation (Hersenstichting) (n=10)  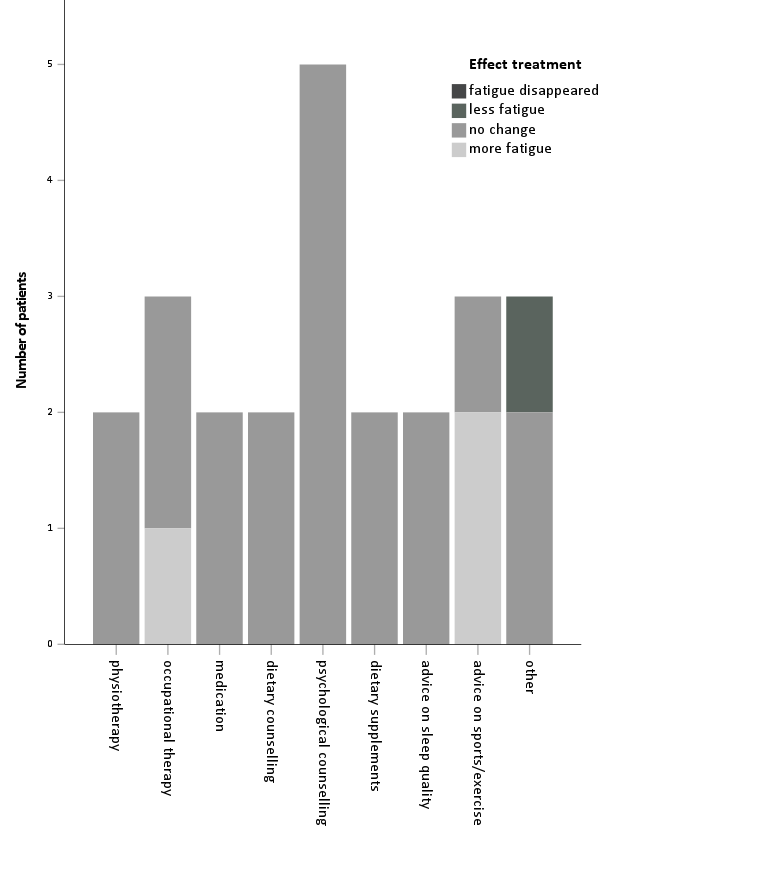 | j) | Dutch Foundation for Mental Health (MIND) (n=11)  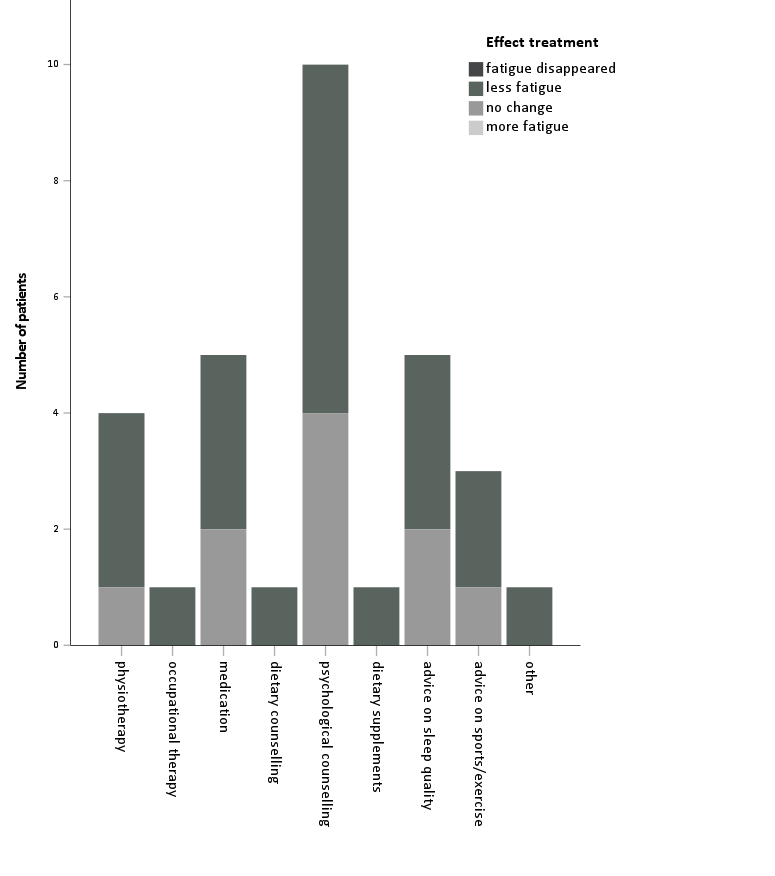 |
| --- | --- | --- | --- |

| k) | Dutch Burn Foundation (Brandwonden Stichting) (n=20)  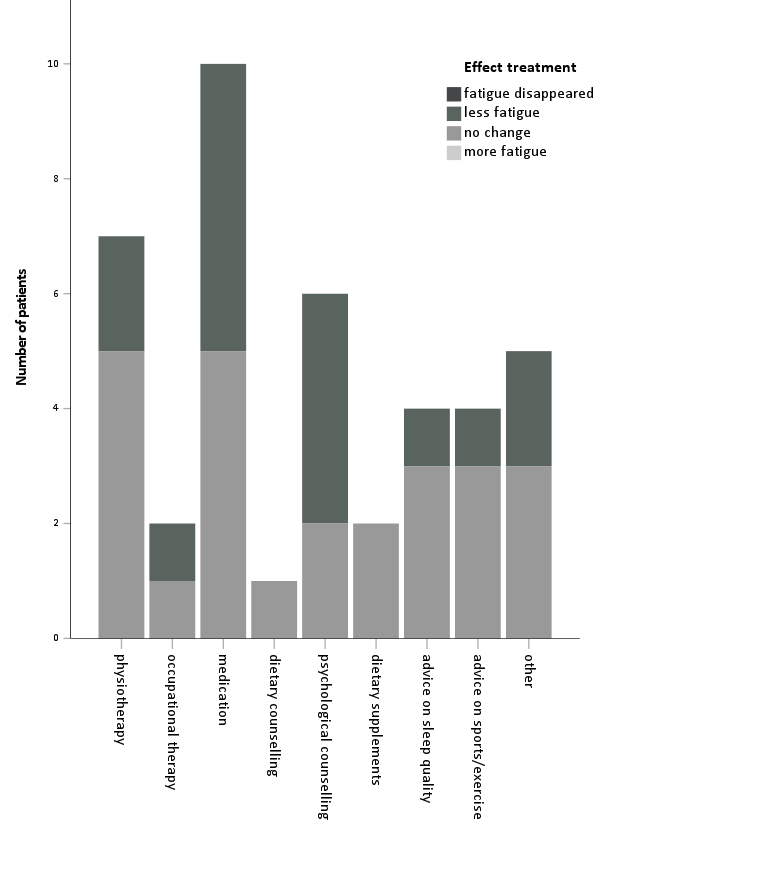 | l) | Dutch ME/CFS Foundation (ME/CVS Stichting) (n=137)  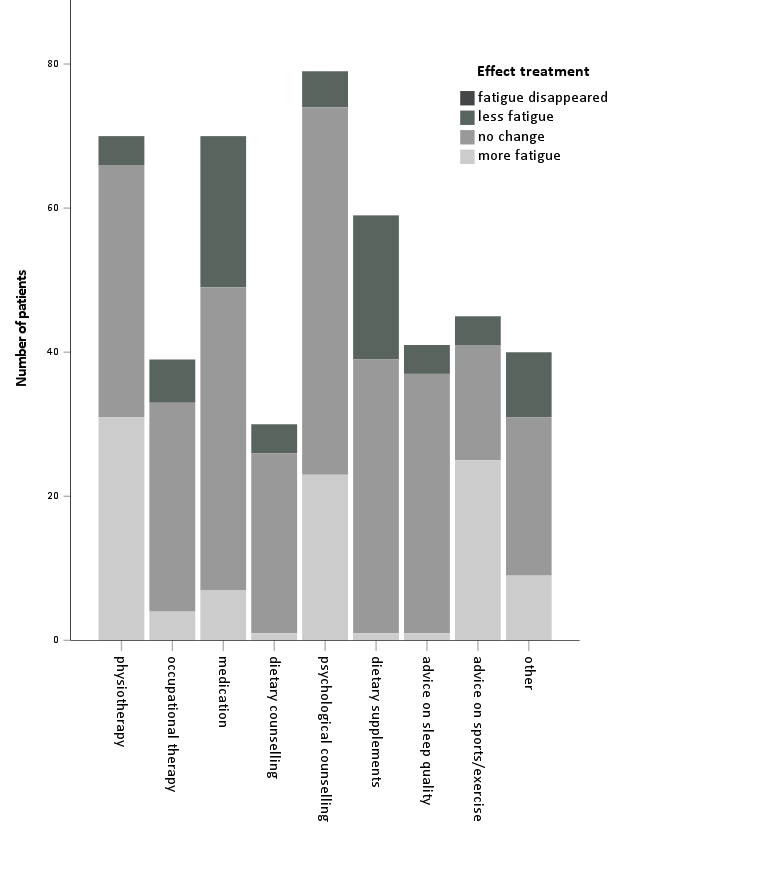 |
| --- | --- | --- | --- |

| m) | Dutch Patient Association for Cardiovascular Diseases (Harteraad) (n=35)  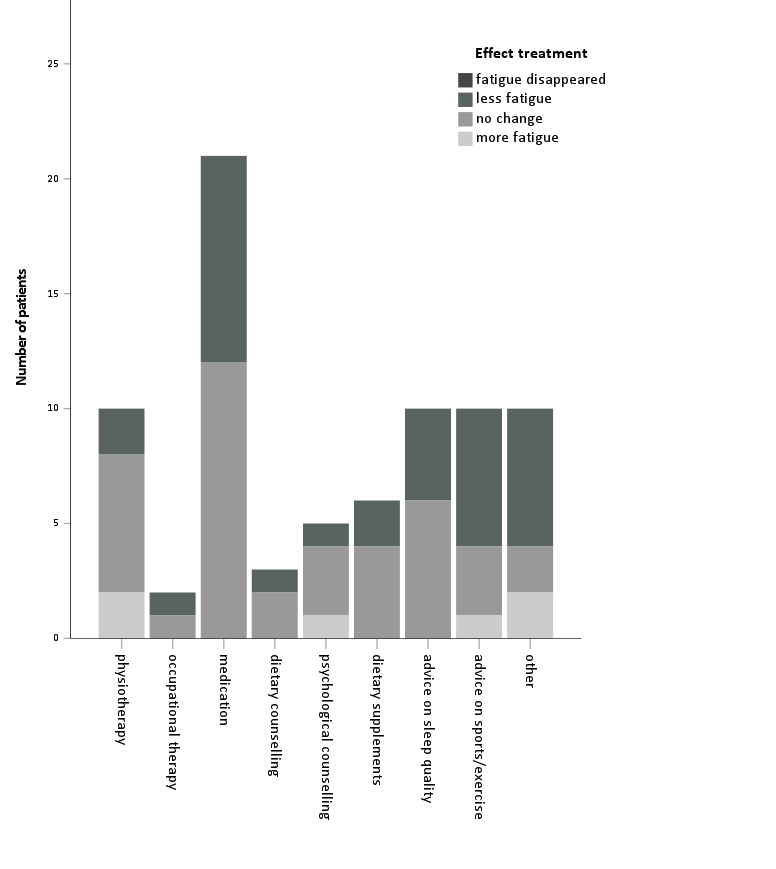 | n) | Irritable Bowel Syndrom Patient Association (Prikkelbare Darm Syndroom Belangenorganisatie) (n=3)  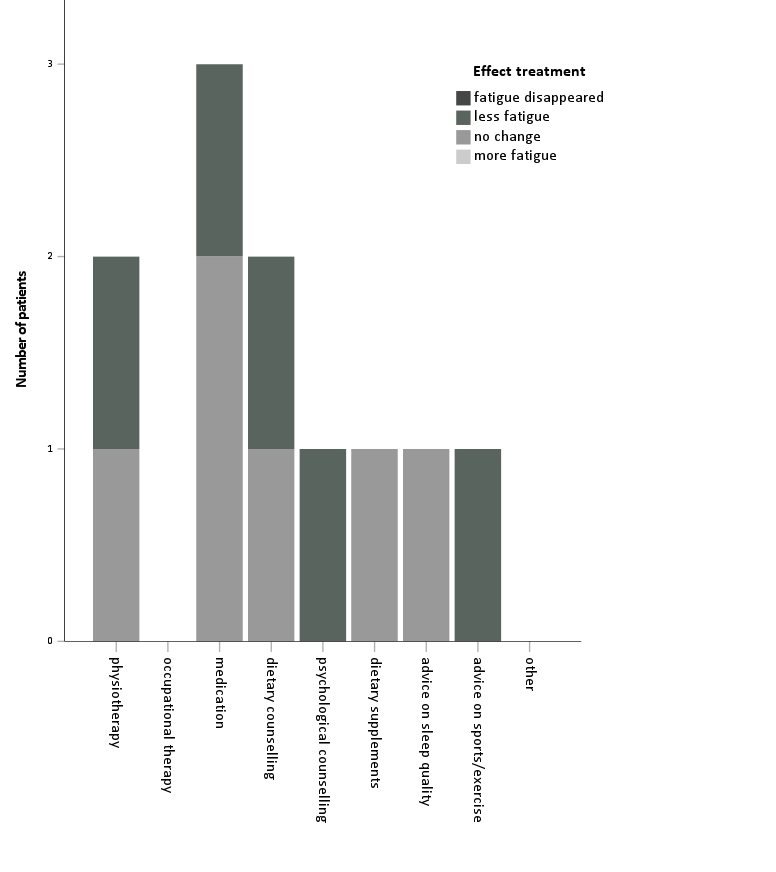 |
| --- | --- | --- | --- |
