# Supplementary material for: Physical and mental fatigue in people with non-communicable chronic diseases
Source: Ann Med. 2022 Sep 16;54(1):2522–34. doi: 10.1080/07853890.2022.2122553 (PMC9487929; doi:10.1080/07853890.2022.2122553)
Supplement: Supplemental Material [file IANN_A_2122553_SM4401.docx]

**Supplemental Figure 1**

Physical and mental fatigue in non-communicable chronic diseases stratified by patient

association

1. Dutch Heart Foundation (Hartstichting) (n=544)
2. Dutch Arthritis Society (ReumaNederland) (n=2,060)
3. Lung Foundation Netherlands (Longfonds) (n=167)
4. Dutch Kidney Foundation (Nierstichting) (n=159)
5. Dutch Diabetes Foundation (Diabetes Fonds) (n=73)
6. Princess Beatrix Muscle Foundation (Prinses Beatrix Spierfonds) (n=58)
7. Dutch Neuromuscular Disease Association (Spierziekten Nederland) (n=213)
8. Dutch Digestive Disease Foundation (Maag Lever Darm Stichting) (n=337)
9. Dutch Brain Foundation (Hersenstichting) (n=28)
10. Dutch Foundation for Mental Health (MIND) (n=43)
11. Dutch Burn Foundation (Brandwonden Stichting) (n=103)
12. Dutch ME/CFS Foundation (ME/CVS Stichting) (n=250)
13. Dutch Patient Association for Cardiovascular Diseases (Harteraad) (n=142)
14. Irritable Bowel Syndrom Patient Association (Prikkelbare Darm Syndroom Belangenorganisatie) (n=22)

**Supplemental Figure 2** Physical and mental fatigue in non-communicable chronic diseases stratified by patient association

| a) | Dutch Heart Foundation  (Hartstichting)  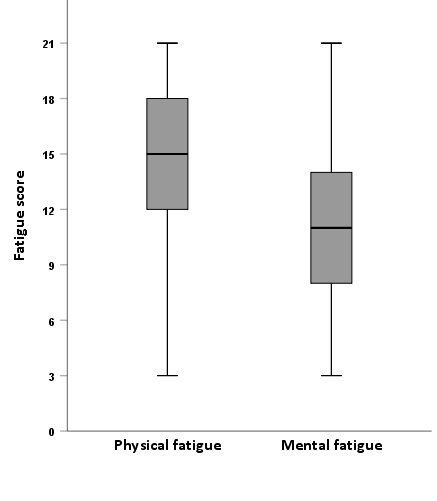 | b) | Dutch Arthritis Society  (ReumaNederland)  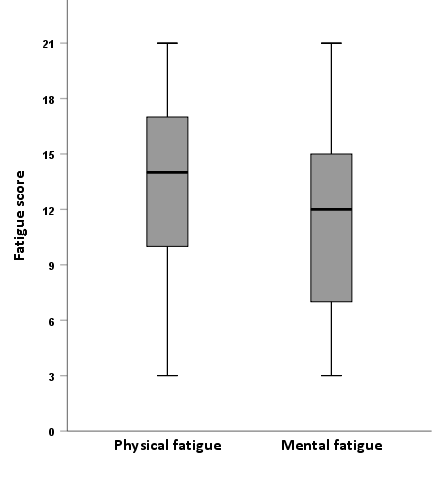 |
| --- | --- | --- | --- |
| c) | Lung Foundation Netherlands  (Longfonds)  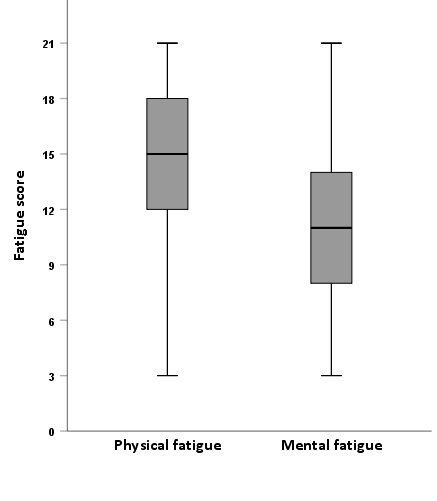 | d) | Dutch Kidney Foundation  (Nierstichting)  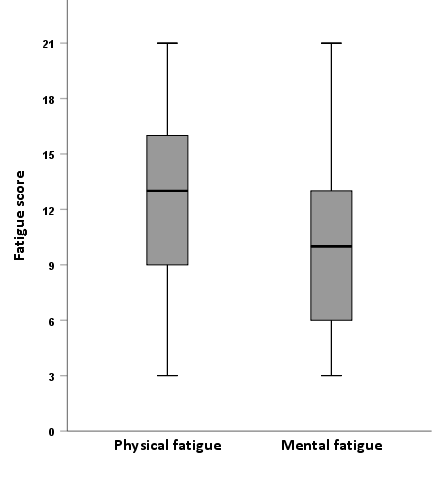 |

| e) | Dutch Diabetes Foundation  (Diabetes Fonds)  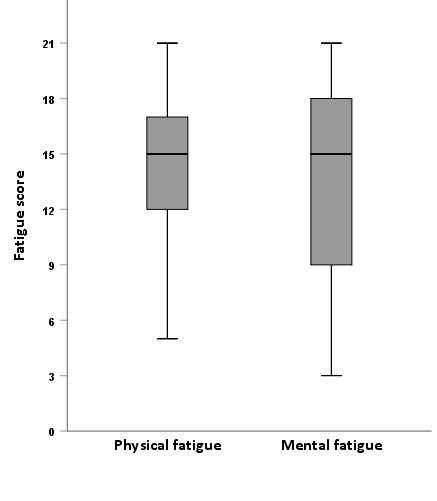 | f) | Princess Beatrix Muscle Foundation  (Prinses Beatrix Spierfonds)  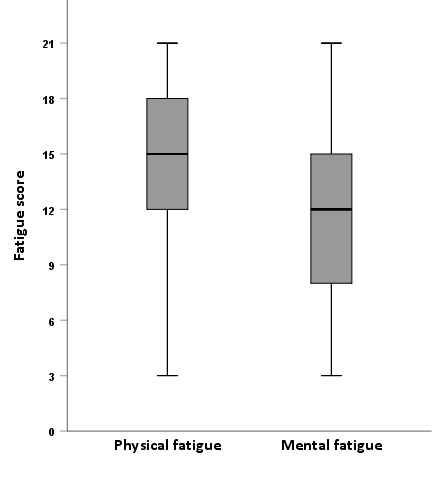 |
| --- | --- | --- | --- |
| g) | Dutch Neuromuscular Disease Association  (Spierziekten Nederland)  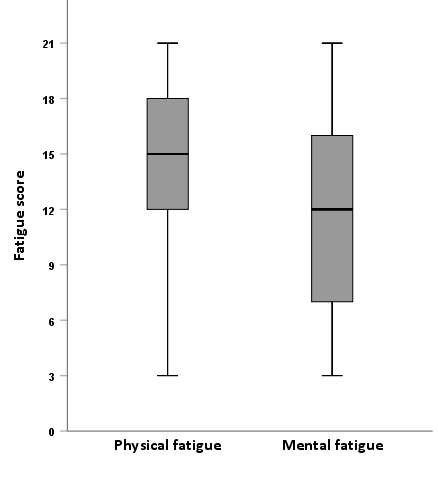 | h) | Dutch Digestive Disease Foundation  (Maag Lever Darm Stichting)  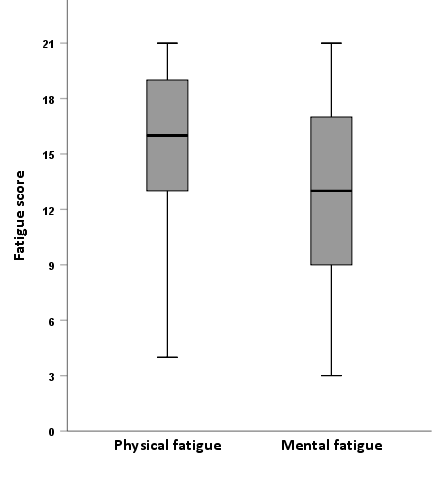 |

| i) | Dutch Brain Foundation  (Hersenstichting)  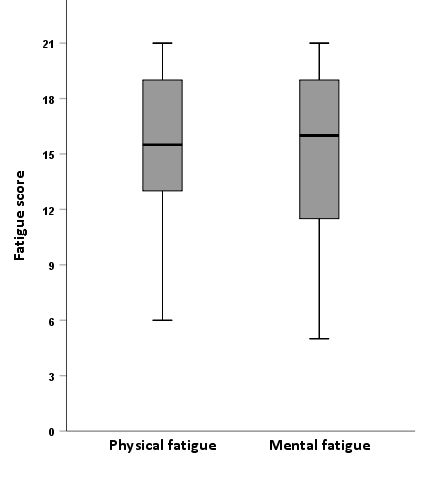 | j) | Dutch Foundation for Mental Health  (MIND)  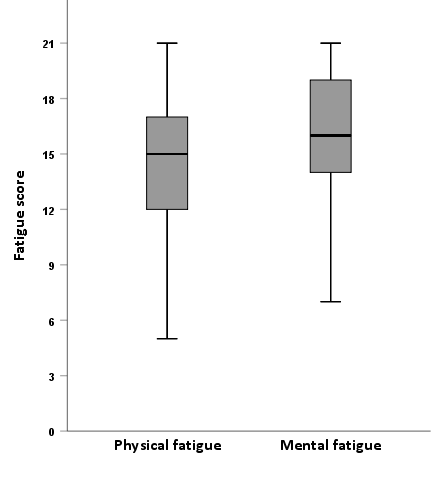 |
| --- | --- | --- | --- |
| k) | Dutch Burn Foundation  (Brandwonden Stichting)  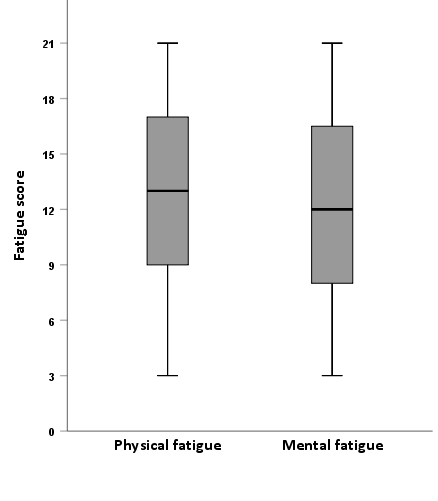 | l) | Dutch ME/CFS Foundation  (ME/CVS Stichting)  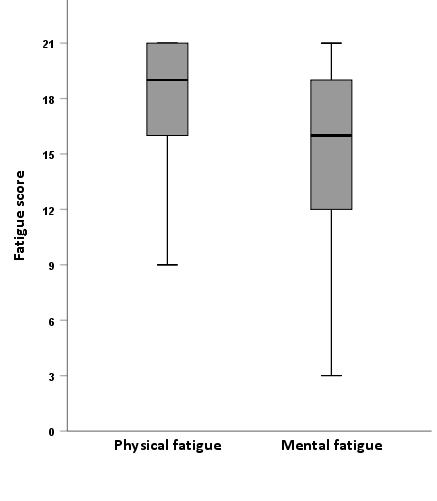 |

| m) | Dutch Patient Association for Cardiovascular Diseases  (Harteraad)  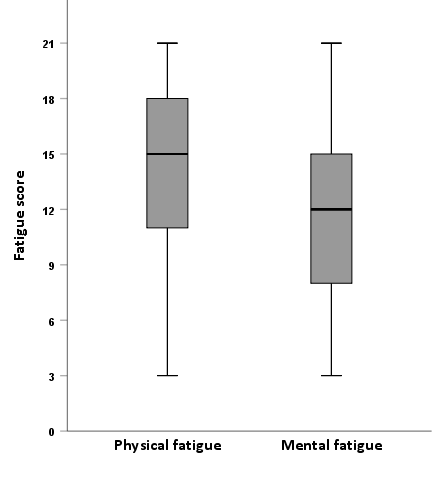 | n) | Irritable Bowel Syndrom Patient Association  (Prikkelbare Darm Syndroom Belangenvereniging)  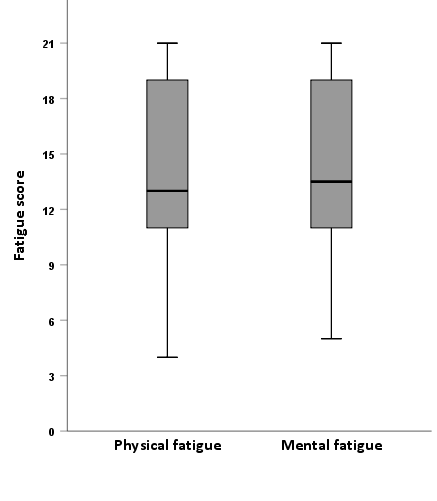 |
| --- | --- | --- | --- |
